# Penile Shaft Abscess: A Rare Urological Occurrence

**DOI:** 10.7759/cureus.105853

**Published:** 2026-03-25

**Authors:** Xiang Yuen Po

**Affiliations:** 1 Urology, Northeast Health Wangaratta, Wangaratta, AUS

**Keywords:** cavernosal abscess, corpus cavernosum abscess, penile abscess, penile shaft infection, urological infection

## Abstract

Penile shaft abscess is a rare urological condition characterized by a localized collection of purulent material within the tissues of the penile shaft. Due to its uncommon occurrence, most knowledge regarding this condition is derived from isolated case reports and small case series. Although uncommon, penile abscesses are clinically significant because delayed diagnosis or inadequate management may lead to complications such as penile fibrosis, curvature, erectile dysfunction, or systemic infection. Prompt recognition and appropriate treatment are therefore essential to ensure favorable functional outcomes. We present a case report of a 37-year-old male who presented with a penile shaft abscess and was re-treated with intravenous antibiotics and surgical drainage. Recovery was unremarkable, and there was no recurrence of abscess on follow-up a month after surgery, and the patient maintained potency without penile deformity on self-report. The etiology, diagnosis, and management of penile shaft abscesses are discussed.

## Introduction

Penile shaft abscess is an uncommon urological condition with limited cases reported in the literature. It has been described in a variety of clinical settings, including penile trauma, intracavernosal injection therapy for erectile dysfunction, penile instrumentation, and priapism. It may also occur in association with underlying systemic conditions such as poorly controlled diabetes mellitus or immunosuppression, which predispose patients to infection. Despite these recognized risk factors, spontaneous cases without clear precipitating events have also been documented, highlighting the variable and often multifactorial etiology of this condition.

The microbiology of penile abscesses typically reflects skin and genitourinary flora, with commonly implicated organisms including *Staphylococcus aureus*, *streptococci*, and anaerobes such as *Bacteroides* [1]. Clinical presentation can vary from localized penile swelling and pain to systemic features of infection, and delayed diagnosis may result in significant complications, including corporal fibrosis, penile deformity, or erectile dysfunction.

Diagnosis is often made clinically but may be supported by imaging modalities such as ultrasound or magnetic resonance imaging, which can help delineate the extent of the abscess and guide management [2]. Early recognition and prompt intervention are essential to optimize outcomes, with treatment typically involving a combination of antimicrobial therapy and surgical drainage.

We present a rare case of penile shaft abscess caused by a trapped hair, an unusual etiology not commonly reported in the literature. This case highlights the importance of considering atypical causes in patients without traditional risk factors and underscores the role of timely surgical management in achieving favorable functional and cosmetic outcomes. A review of the current literature is also provided.

## Case presentation

A 37-year-old male presented to the emergency department (ED) with a one-week history of penile swelling and increasing pain. Patient denies any recent trauma, urinary tract infection, sexually transmitted disease, or intravenous drug use. He also does not have any past medical history or take any regular medications.

**Figure 1:**
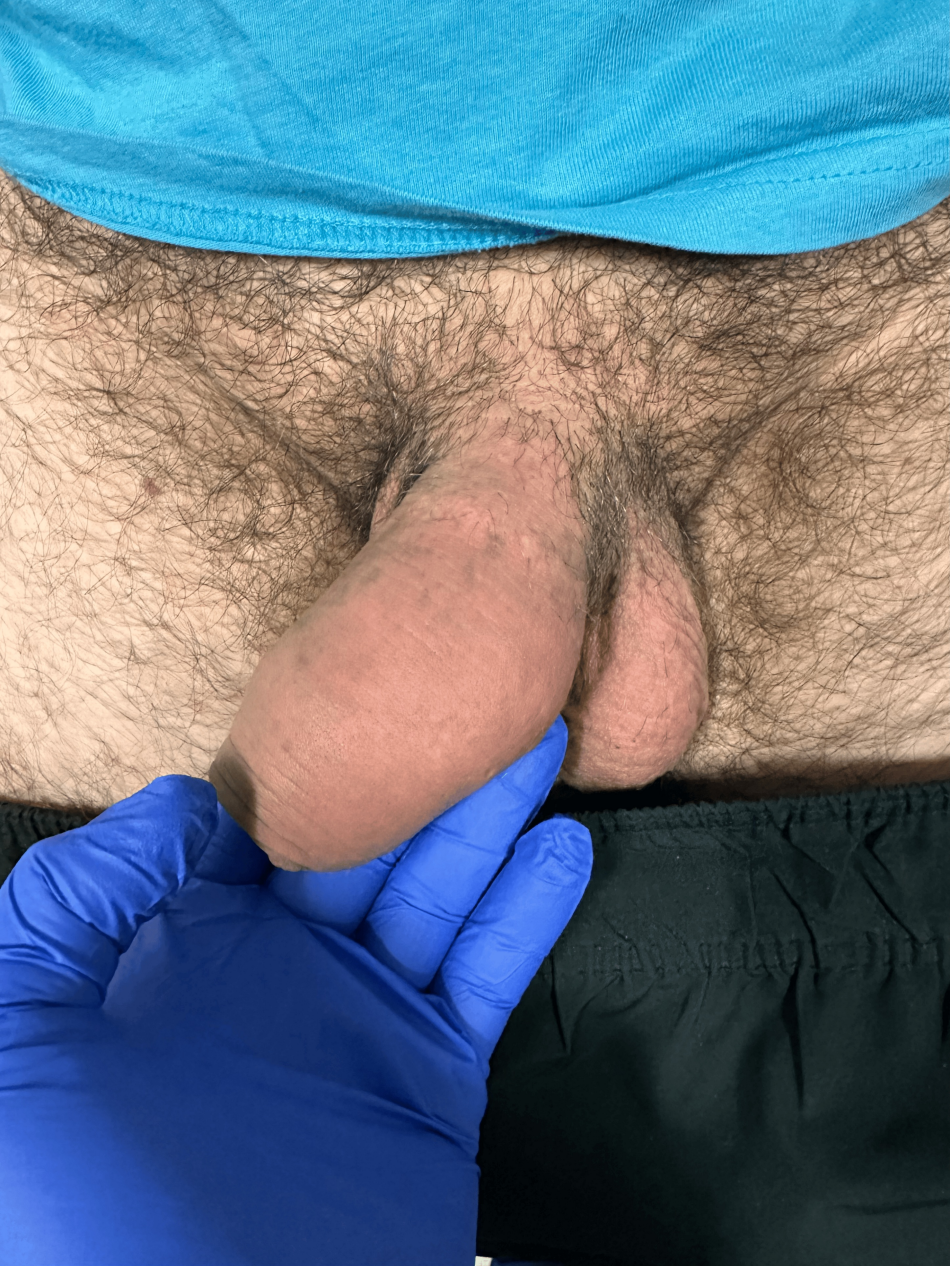
Penile abscess

On presentation to the ED, he was hemodynamically stable and afebrile. On examination, there is a tender, fluctuant area about 6 x 5 cm over the left corpus cavernosum region with surrounding erythema and warmth (Figure 1). There was no discharge from the fluctuant area or penile discharge on expression. His testicular examination was unremarkable, and there was no surrounding penile lesion that would indicate infection by sexually transmitted disease (Figure 1).

Investigations

On admission, the blood tests showed an elevated white cell count and C-reactive protein, and a normal blood glucose (Table 1). Urinalysis on presentation showed no leukocytes or nitrites in his urine. Urine was sent off for microscopy and culture, it returned negative for any growth including sexually transmitted disease. No imaging modality was utilised in this case, as the diagnosis was made clinically.

**Table 1:** Laboratory test findings CRP: C-reactive protein

Test	Patient’s value	Normal reference range
White Cell Count	16x10^9/L	4-11x10^9/L
CRP	167 mg/L	<10 mg/L
Blood glucose	6.6 mmol/L	4-7 mmol/L

Treatment

**Figure 2:**
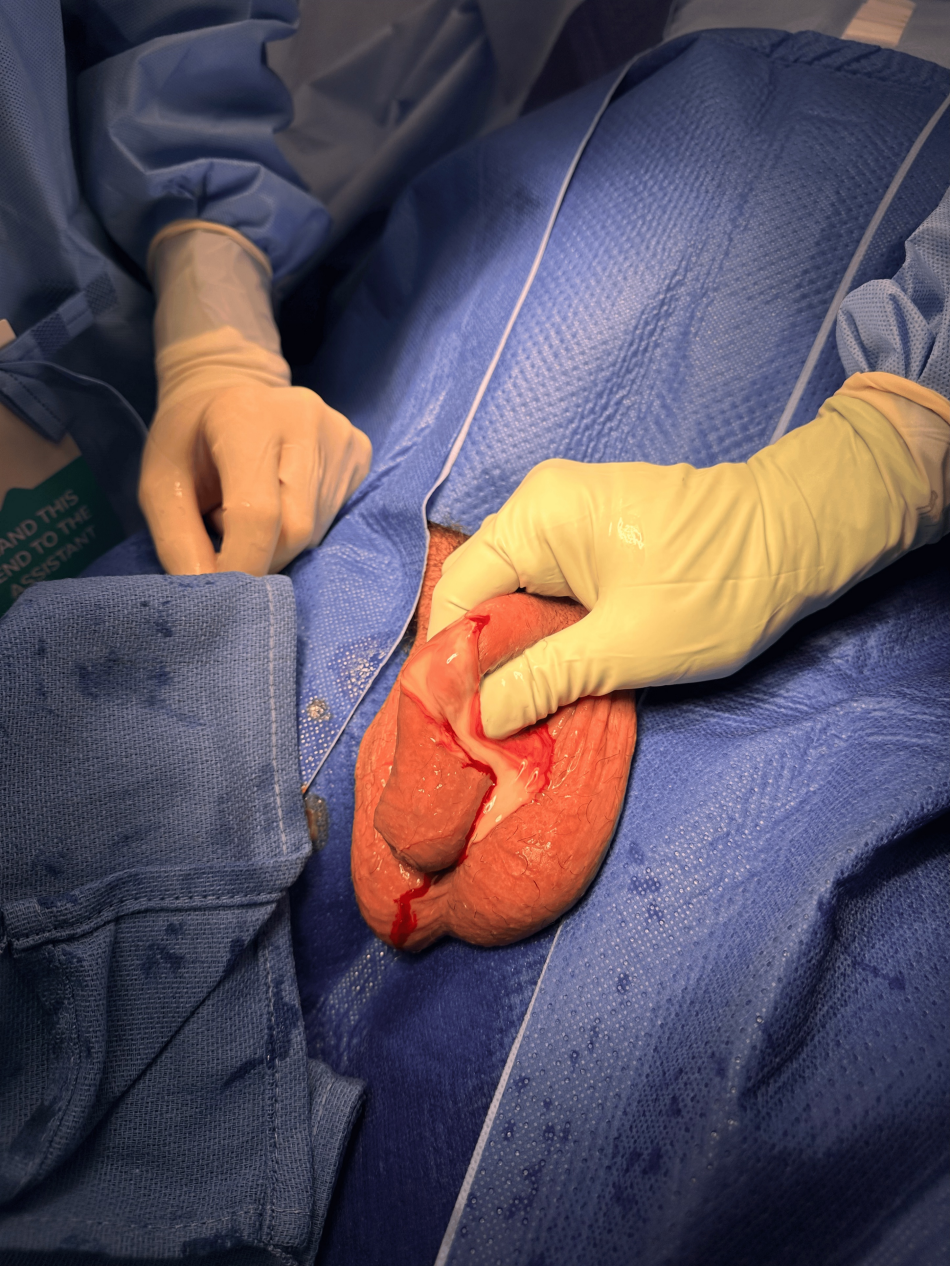
Purulent discharge from penile abscess

Once the diagnosis was made, the patient was commenced on intravenous antibiotics, flucloxacillin to cover skin sources of abscess, and proceeded to surgical intervention under general anesthesia. Intraoperatively, the abscess was identified as superficial and not involving the corpus cavernosum. An incision was made over the most fluctuant area of the penile shaft, allowing drainage of approximately 10 mL of purulent material (Figure 2). The tunica albuginea was not entered. Pus was sent for microscopy and culture. 

Following drainage, the abscess cavity was thoroughly irrigated with copious saline washout. A hair was identified within the abscess cavity, raising the possibility of a foreign body acting as the nidus for infection. No surgical drain was placed. The cavity was packed with Kaltostat, which was removed on postoperative day one.

Postoperatively, the patient was managed with simple penile dressings and continued antibiotics. He was subsequently discharged with instructions for routine wound care and a further course of oral flucloxacillin for a week. 

A week after discharge, he was seen in the clinic for a wound check, and the healing was satisfactory. Pus-cultured mixed skin flora without any specific microorganism grown. Patient maintained potency without penile deformity on self-report (Figure 2).

## Discussion

A penile abscess is an uncommon urological condition characterized by a localized purulent collection within the penile soft tissues, most frequently involving the corpus cavernosum or, less commonly, the corpus spongiosum. Reported etiologies include trauma, intracavernosal injection for erectile dysfunction, extension of peri-urethral infection, sexually transmitted disease, and iatrogenic causes such as instrumentation and spontaneous, which is rare in otherwise healthy individuals [1]. Although rare, it carries potential for significant morbidity, including erectile dysfunction, fibrosis, and deformity if not promptly diagnosed and treated.

The pathogenesis typically involves bacterial inoculation into the vascular sinusoids of the corpora or along fascial planes. The most frequently isolated organisms include *Staphylococcus aureus*, *Streptococcal* species, *Escherichia coli*, *Bacteroides*, and polymicrobial flora [3]. Risk factors such as diabetes mellitus, intravenous drug use, and immunosuppression increase susceptibility [4]. 

Clinically, patients usually present with penile swelling, erythema, fluctuance, pain, and sometimes fever or systemic inflammatory response [1]. Painful erections or priapism-like symptoms may occur if the corpus cavernosum is involved. Differential diagnoses include cellulitis, Fournier’s gangrene, Peyronie’s plaques, and penile tumors. Ultrasound is a useful initial modality for identifying fluid collections, while MRI offers superior delineation of abscess extent, sinus tracts, and cavernosal involvement [5]. 

Surgical drainage remains the gold standard, particularly for cavernosal abscesses, and should be accompanied by systemic antibiotic therapy [4]. While in this case the abscess was located in the subcutaneous tissue, its management remains the same as that of a cavernosal abscess. Minimally invasive approaches, including ultrasound-guided percutaneous drainage, have been successfully reported and may reduce morbidity when infection is localized [6]. Empiric antibiotic coverage should target gram-positive cocci and gram-negative facultative anaerobes until cultures guide therapy. Delayed or inadequate treatment increases the risk of corporal fibrosis, erectile dysfunction, and penile curvature [1].

Outcomes are generally favorable with timely intervention. However, long-term sequelae such as erectile dysfunction or penile deformity can occur, especially when cavernosal tissue has been extensively involved. Early urological consultation, optimal imaging, culture-guided antimicrobial therapy, and appropriate surgical technique are essential to reduce complications. Given the rarity of penile abscesses, most available literature comprises case reports and small series, highlighting the need for increased awareness and early diagnostic consideration in patients presenting with atypical penile pain and swelling.

## Conclusions

Penile shaft abscess is a rare urological entity that requires a high index of suspicion, particularly in patients without classical risk factors. This case demonstrates an unusual etiology, with a trapped hair likely acting as a nidus for infection, highlighting that foreign bodies should be considered in atypical presentations. Although imaging can aid in diagnosis, careful clinical assessment remains central, particularly in differentiating superficial from cavernosal involvement, which has implications for surgical planning.

Definitive management with prompt incision and drainage combined with appropriate antimicrobial therapy remains the cornerstone of treatment, regardless of abscess location. Early intervention is critical to prevent complications such as corporal fibrosis, penile deformity, and erectile dysfunction. This case further demonstrates that favorable functional outcomes can be achieved with timely and appropriate management, even in uncommon presentations.

Given the limited number of reported cases, this report contributes to the growing body of literature by highlighting a rare cause and reinforcing key principles in diagnosis and management. Increased awareness of atypical etiologies and adherence to prompt surgical management are essential to optimize patient outcomes.
